# Single-cycle surface plasmon polaritons on a bare metal wire excited by relativistic electrons

**DOI:** 10.1038/ncomms13769

**Published:** 2016-12-23

**Authors:** W.P.E.M. op ‘t Root, G.J.H. Brussaard, P.W. Smorenburg, O.J. Luiten

**Affiliations:** 1 Coherence and Quantum Technology, Department of Physics, Eindhoven University of Technology, Eindhoven 5600 MB, The Netherlands; 2 Institute for Complex Molecular Systems, Eindhoven University of Technology, Eindhoven 5600 MB, The Netherlands

## Abstract

Terahertz (THz) pulses are applied in areas as diverse as materials science, communication and biosensing. Techniques for subwavelength concentration of THz pulses give access to a rapidly growing range of spatial scales and field intensities. Here we experimentally demonstrate a method to generate intense THz pulses on a metal wire, thereby introducing the possibility of wave-guiding and focussing of the full THz pulse energy to subwavelength spotsizes. This enables endoscopic sensing, single-shot subwavelength THz imaging and study of strongly nonlinear THz phenomena. We generate THz surface plasmon polaritons (SPPs) by launching electron bunches onto the tip of a bare metal wire. Bunches with 160 pC charge and ≈6 ps duration yield SPPs with 6–10 ps duration and 0.4±0.1 MV m^−1^ electric field strength on a 1.5 mm diameter aluminium wire. These are the most intense SPPs reported on a wire. The SPPs are shown to propagate around a 90° bend.

## Abstract (web summary)

Here, the authors demonstrate how ultra-short bunches of relativistic electrons produce coherent transition radiation at the tip of a thin wire. The radiation then propagates as a powerful surface plasmon polariton along the wire, illustrating the potential of this technique for terahertz plasmonics.

The terahertz (THz) part of the electromagnetic spectrum (0.1–10 THz) is ideal for probing and manipulating fundamental excitations in solids and rotational states in molecules. The development of THz techniques in recent years has led to many exciting applications, such as medical imaging, characterization of materials, security screening and industrial process control. In particular, the development of high-intensity, pulsed THz sources based on rectification of femtosecond laser pulses has revolutionized THz science and technology^1^,^2^,^3^,^4^,^5^. Even more spectacular progress in this vibrant field is expected from the development of THz plasmonics, presently a very active area of research, enabling new ways to control and manipulate THz radiation^6^,^7^.

By providing guided delivery of intense THz pulses, THz waveguides^8^ open up new opportunities in photonics, such as single-shot, subwavelength THz imaging and possibly new applications based on nonlinear THz phenomena^9^. In cancer research, endoscopic systems based on wave-guiding intense THz pulses could greatly extend THz cancer detection schemes, which are today limited to tissue that can be reached with optical techniques^10^,^11^. By tapering a THz wire waveguide into a tip, the THz pulses can be focussed to subwavelength spot-sizes, which further increases the intensity^12^,^13^,^14^,^15^,^16^,^17^,^18^,^19^,^20^. One interesting application of this is a new class of pulsed electron guns that use the wire tip as an electron emitter with record brightness^21^. Moreover, the subwavelength focussing of THz pulses provided by tapered wire waveguides greatly improves the spatial resolution of THz experiments^12^,^13^,^14^,^15^,^16^,^17^,^18^,^19^,^20^. In recent years, several methods have been devised to localize free-space THz radiation to nanometre-sized structures such as atomic force microscopic tips^22^ and nanolithography-based slits^23^, in which a small fraction of the THz pulse is locally enhanced to the kV cm^−1^ range at the sample with spatial resolutions in the nanometre range. Even subcycle temporal resolution of 10 fs has been achieved based on gated, multi-shot measurements using multicycle THz pulses^22^. This fascinating field can be further developed by using strong subcycle THz pulses that are wire-guided, which enables focussing of the full THz pulse energy to subwavelength distances, while providing temporal resolution through the subcycle THz pulse length. This will enable single-shot subwavelength THz imaging and the study of strongly nonlinear THz phenomena^9^.

Accelerator-based systems and optical techniques exist to produce short intense THz pulses propagating in free space^1^,^2^,^3^,^4^,^5^,^24^,^25^,^26^. Such THz pulses have been coupled onto wire waveguides where they propagate as surface plasmon polaritons (SPPs) along the surface of the wire^8^,^27^,^28^,^29^,^30^,^31^,^32^. Unfortunately, creating SPPs by coupling free-space THz radiation onto a metal wire is inefficient owing to the very poor spatial overlap between the linearly polarized free-space mode and the radially polarized waveguide mode^28^,^29^,^31^,^32^. Additionally, direct SPP excitation techniques involving electron beams interacting with flat surfaces have been demonstrated^33^ but not on a wire. In 2008, we proposed a method in which ultrashort bunches of relativistic electrons generate coherent transition radiation (CTR) at the tip of a thin wire^34^. The radiation produced then propagates as a powerful SPP along the wire, which serves as a waveguide. This hybrid technique integrates accelerator-based CTR methods and plasmonic techniques to produce intense subcycle pulses in the THz frequency range directly on a bare metal wire waveguide^34^. By this method, extremely intense and highly localized THz pulses can be created using state-of-the-art table-top-sized accelerators^35^. The GeV electron bunches currently driving X-ray free electron lasers (X-FELs)^25^ would enable nonlinear ‘THz pump—X-ray probe' experiments in which micron-sized pieces of material are excited by extreme THz field strengths and subsequently probed by X-ray pulses to study structural dynamics at femtosecond timescales.

The proposed method relies on the natural match of the Coulomb field of relativistic electron bunches with the radially polarized electric field of the guided surface wave. Firing electron bunches onto the tip of a tapered metal wire causes the radially polarized field of the electron bunches to transfer to the wire. This results in SPPs propagating along the wire. The physical mechanism behind this process can be understood using a field line model as described by Purcell^36^, which is described in the Methods section. From this model, the estimated peak electric field strength *E* of the SPP generated on the wire is given by


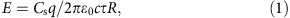


where *q* is the electron bunch charge, *τ* the bunch duration, *R* the radius of the wire, *ɛ*_0_ the vacuum permittivity and *c* the speed of light. The numerical dimensionless constant *C*_s_ takes the three-dimensional bunch shape into account and depends mildly on the beam energy; *C*_s_ can be calculated using more elaborate methods^34^. For ellipsoidal bunches with a length *cτ* (semiaxis) and 2 MeV energy, *C*_s_≈0.7. Meanwhile, the length of the SPP is comparable to that of the generating electron bunch, with a spectrum coherent up to frequencies ∼1*/τ*.

Here we experimentally demonstrate the method proposed in ref. 34. By firing electron bunches with an energy of 3.1 MeV, bunch charge *q=*160 pC and full-width-at-half-maximum (FWHM) duration of *τ* ≈6 ps on a sharp conical tip of an aluminium wire with a diameter of 1.5 mm, we generate SPPs with a FWHM duration of 6–10 ps and electric field strength of 0.4±0.1 MV m^−1^ on the surface of the wire. This yields the possibility of wave-guiding and subwavelength focussing of the full THz pulse, enabling endoscopic delivery and study of strongly nonlinear THz phenomena. Equation (1) shows that the SPP field strength on the wire is proportional to the peak electron beam current ∼*q*/*τ*. Using the 100 fs, 100 pC, 3.7 MeV bunches that can be produced in a table-top radio frequency photogun setup comparable to ours^35^, THz field strengths of tens of MV m^−1^ could be generated on a millimetre-diameter wire. A fortiori, the sub-100-fs, nanocoulomb electron bunches driving X-FELs have the potential to generate THz pulses on a millimetre-diameter wire in excess of 1 GV m^−1^, comparable to the record field strengths obtained by free-space CTR at Stanford Linear Accelerator Center (SLAC)^25^.

## Results

### Experimental setup

**Figure 1:**
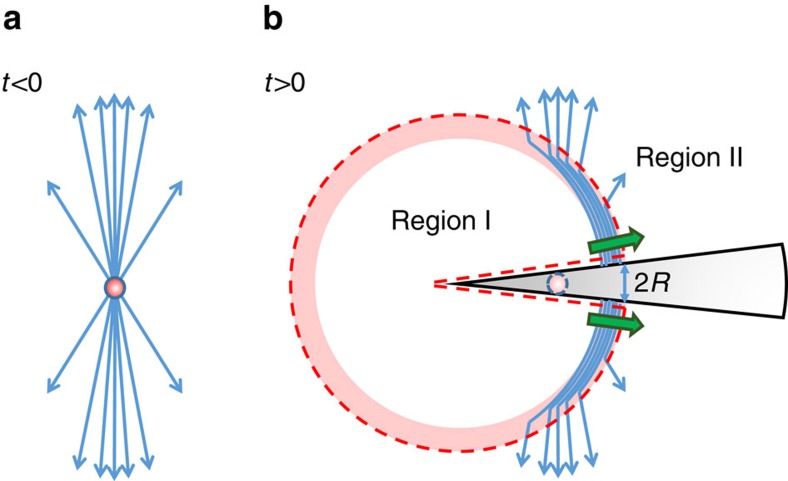
Field line model of SPP formation by firing an electron bunch onto a wire. (**a**) Electric field lines of the electron bunch before impact. (**b**) Field lines shortly after impact. The dashed red line indicates the sphere with radius *cτ* and conical cut-out (that is, the Gauss volume used in the text). Inside region I, the electric field of the electron bunch is completely screened by the metal wire. The field in region II is that of an undisturbed electron. At the boundary between the two regions, a thin shell of dense field lines is formed (shaded band). The thick green arrows indicate the concentration of field lines forming an SPP that propagates onto the wire.

**Figure 2:**
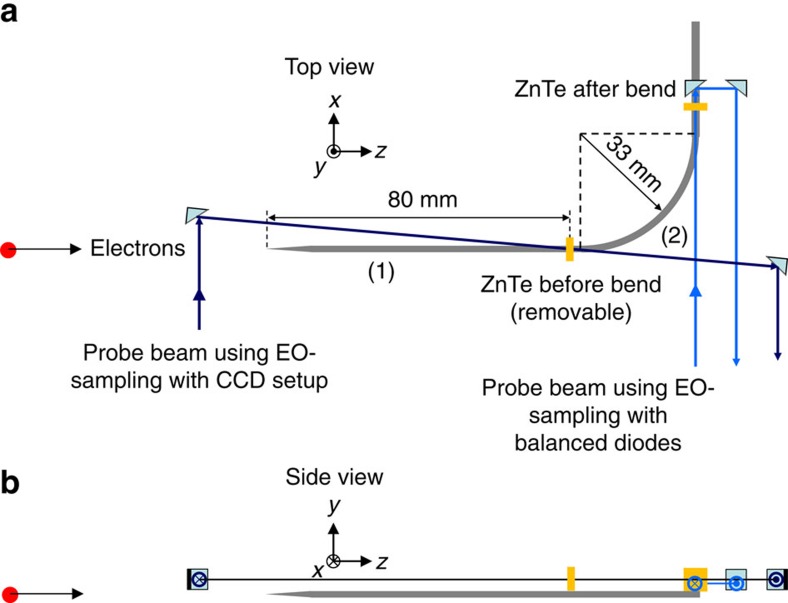
Experimental layout. (**a**) Top view of the used experimental layout. (**b**) Side view of the used experimental layout. When an electron bunch hits the tip of the wire, part of the bunch is absorbed, generating a surface wave propagating along the wire. When the surface wave passes the first ZnTe crystal at the end of the straight section (1), it induces birefringence in the crystal that changes the polarization of the probing laser pulse. After reflection on a turning mirror, the laser pulse exits the chamber and is imaged onto two CCD cameras (not shown here) to obtain the image shown in Fig. 3a. To measure the field strength of the surface wave after propagating around the bent section (2) in the wire, the first ZnTe crystal (before the wire bend) is removed. In this configuration, the probing laser pulses pass through the second ZnTe crystal and are analysed using two photodiodes to measure the change in polarization. By varying the delay between the electron bunches and the laser pulses, the time dependence of the electric field of the surface wave is obtained, shown in Fig. 4. The coordinate system shown in this figure is used throughout the paper.

The basic principle of using an ultrashort bunch of relativistic electrons to produce CTR at the tip of a thin wire, which subsequently propagates along the wire as a powerful SPP, is illustrated in Fig. 1 and is explained in more detail in the Methods section. We have demonstrated the method in a small-scale experimental setup, which is schematically illustrated in Fig. 2. An animation of the experiment (Supplementary Movie 1) can be found in Supplementary Information. Electron bunches from a 3.1 MeV accelerator containing 160 pC of charge are focussed onto the conical tip of an aluminium wire. The radius *R* of the wire is 0.75 mm and the conical tip has an opening angle 2*δ*=8°. At the tip of the wire, the electron bunches have a radius of 0.2 mm (root mean square (RMS)). When the electrons hit the wire, they are absorbed into the wire, setting up a pulse of transition radiation that propagates along the surface of the wire as an SPP. This surface wave is analysed at the end of the straight part (1) of the wire (see Fig. 2), 80 mm from the tip, by placing a ZnTe <110> crystal (5 × 5 × 0.5 mm^3^) directly on the surface of the wire. A circularly polarized pulse (50 fs, ∼1 μJ, 800 nm) from the Ti:Sapphire laser system is used to probe the crystal. These probe pulses come from the same laser system that is used to generate the electrons through photo-emission in the accelerator and are therefore synchronous with the electron bunches and the SPP. The ZnTe crystal is imaged through a polarizer cube onto two charge-coupled device (CCD) cameras. By taking the relative difference of the two images [(*A*−*B*)/(*A*+*B*)], an image of the field strength of the surface wave in the ZnTe crystal is obtained. Just behind the ZnTe crystal, the wire forms a bend, with a radius of curvature of 33 mm. After the bent section (2) of the wire, a second ZnTe crystal is placed on the wire to determine the field strength of the SPP at this position. Because the electric field of the SPP is weaker after the bend, we do not image this crystal but instead use a balanced diode setup to record the change in polarization of the probe pulses induced by the SPP in the ZnTe crystal. By varying the path length of the probe pulses with respect to the photo-emission pulses, a time scan of the signal is formed^37^,^38^,^39^,^40^.

### SPP measurements before the bend

**Figure 3:**
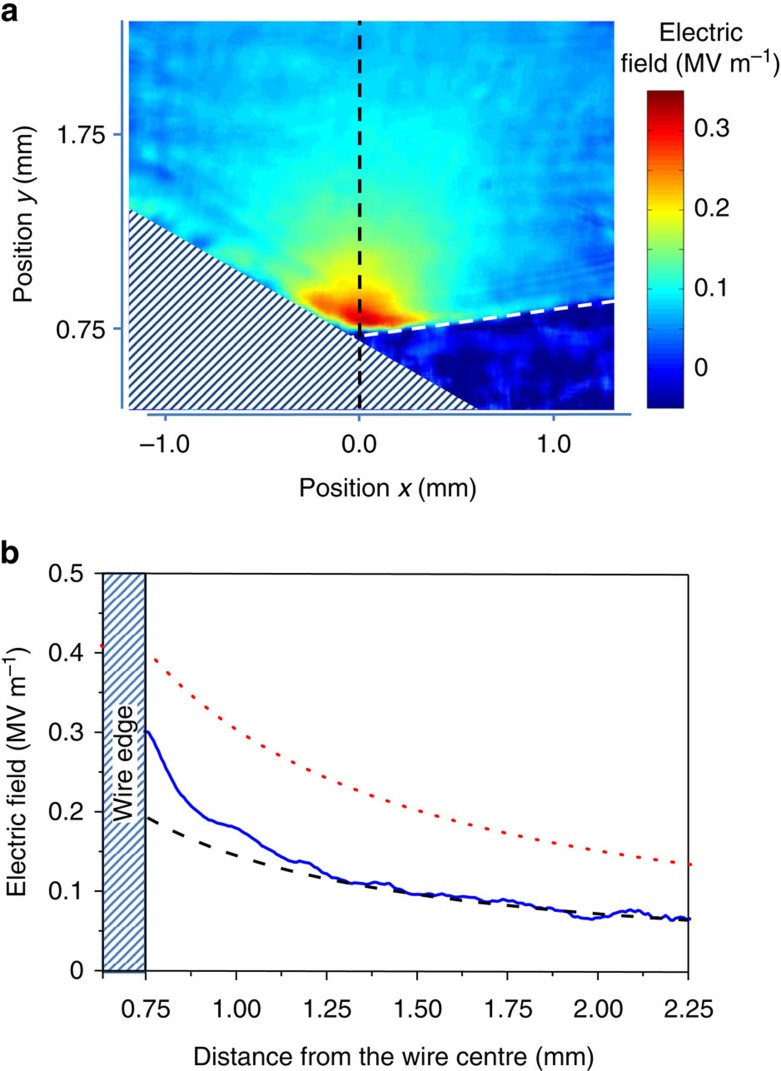
Field strength of the SPP at 80 mm from the tip of the wire. (**a**) Image of the ZnTe crystal touching the wire surface. The colour code represents the relative intensity of the laser pulse passing through the crystal and an analyser and is a measure of the change in polarization caused by the birefringence induced in the crystal by the *y*-component of the electric field of the SPP. The hatched region is the shadow of the wire; the dashed line indicates the edge of the crystal. (**b**) Line-out along the vertical axis *x*=0 of (**a**) (blue solid line). The black dashed line shows the expected *r*^−1^ dependence of the electric field strength at distances greater than *y*=1.25 mm (0.5 mm from the surface of the wire). This line is extrapolated to show the field enhancement near the surface of the wire caused by the mismatch between the permittivity of the crystal and vacuum. The red dotted line is the calculated field strength of the SPP in the absence of the crystal.

Fig. 3a shows the image of the first ZnTe crystal, illuminated by the probe pulse at the moment the SPP passes the crystal. The probe pulse grazes the wire to allow imaging of the point at which the crystal touches the wire. As the probe pulse comes in at a slight angle in the *x* direction (see Fig. 2) and the *y* direction (not shown in Fig. 2), this causes the wire to shade off the lower left corner of the crystal. The relative difference between the intensities reaching both cameras is proportional to the vertical component of the electric field of the SPP inside the ZnTe crystal. The procedure that we used to calculate the absolute value of the electric field and colour code the image of Fig. 3a is described in the Methods section. The image was taken at the maximum field strength of the SPP. The line-out along the vertical axis at *x*=0 is plotted in Fig. 3b. For a cylindrically symmetric SPP on a metal wire with radius much larger than the skin-depth, one expects the radial component of the electric field to fall off as *r*^−1^ (ref. 36, 41), with *r* the radial distance from the wire centre. However, the ZnTe crystal, with a dielectric constant of approximately 10 for the relevant frequencies, artificially enhances the field near the wire surface owing to refraction. We modelled this effect using CST Microwave Studio. At *r*≥2*R*, the electric field shows the expected *r*^−1^ dependence in both the measurement of Fig. 3b and the CST simulation. To extract the undistorted field from the measurements, we therefore fit the data between *y*=1.25 mm and 2.75 mm with this *r*^−1^ dependence and extrapolate to the wire's edge at *y*=0.75 mm (dashed line in Fig. 3b). Finally, we correct for the transmission coefficient of the crystal (*t*_ZnTe_=0.48 at 1 THz) to calculate what the electric field of the SPP would have been without the crystal present. This is shown as the dotted line in Fig. 3b. We thus find that SPPs have been generated directly on a wire by relativistic electron bunches and that the maximum field strength of the SPPs was 0.4±0.1 MV m^−1^.

**Figure 4:**
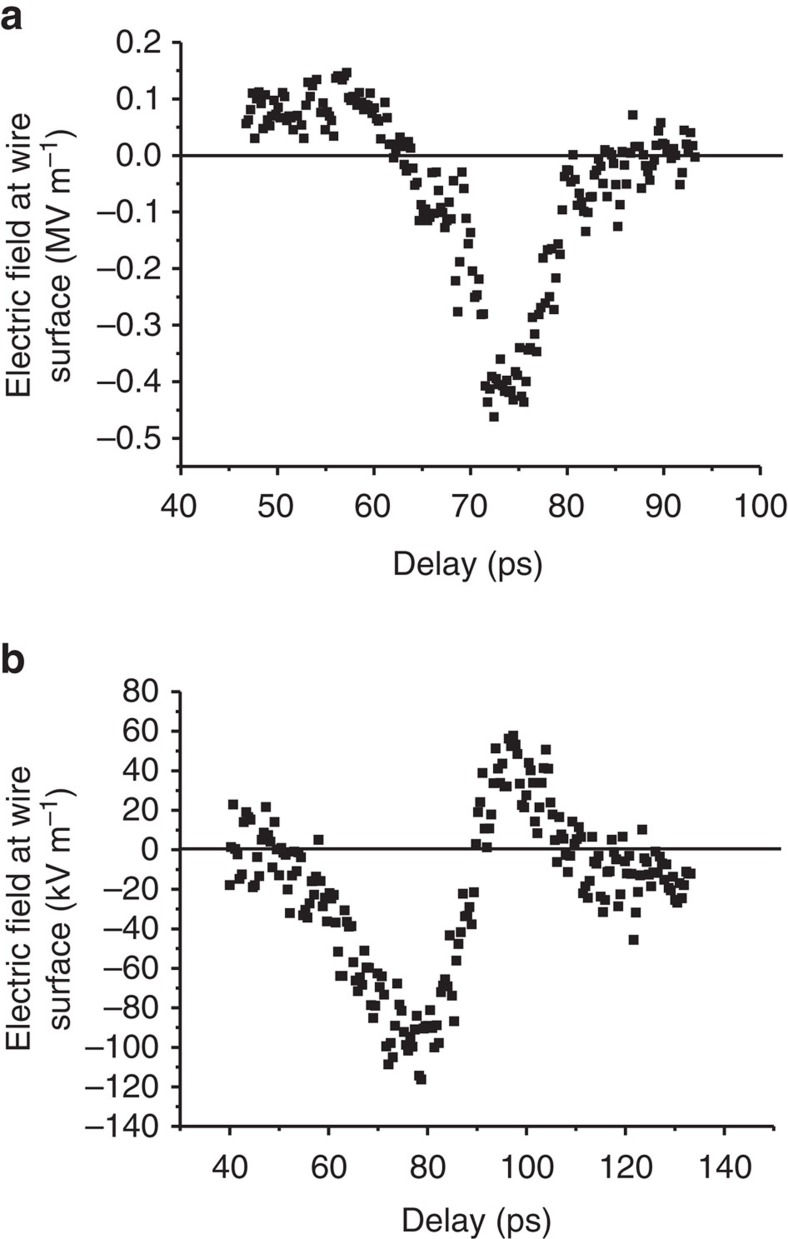
Electric field of the SPP at the surface of the wire as a function of time. (**a**) Measurements of the field strength on the wire surface taken at the position *z*=80 mm from the tip of the wire. For each setting of the time delay between the probe pulse and the laser pulse used for photoemission of the electron bunch, the electric field strength at the surface of the wire was extracted from images recorded in the same manner as Fig. 3. (**b**) Field strengths measured after the bend in the wire (33 mm radius of curvature) using a balanced diode set-up instead of the double-CCD camera used in (**a**). Zero delay is arbitrary for both (**a**,**b**).

By changing the delay between the photo-emission laser pulse and the probe pulse through the ZnTe crystal, we can now determine the field strength on the wire surface as a function of time. The result of such a scan is shown in Fig. 4a. The RMS length of the pulse is between 2 and 3 ps, depending on the level of the baseline that has an uncertainty of ±0.1 MV m^−1^. The signal does not quite return to zero for short delay times, which may be a result of drift in the calibration of the electro-optical system. We are therefore cautious to put a more precise number on the measured pulse duration.

### SPP measurements after the bend

To demonstrate that the SPP is guided by the wire, we have also measured the electric field strength after the 90° bend in the wire using the second ZnTe crystal (see Fig. 2). The laser probe pulses were focussed on the crystal with a spot size of 120±20 μm (FWHM), at a distance of 1.0±0.8 mm from the edge of the wire. During these measurements, the crystal before the bend was removed to avoid disturbing the SPP. Using the same *r*^−1^ scaling as in the imaging technique above, we can calculate the undisturbed field on the surface of the wire in order to compare it with the field strength of the SPP before the bend. The results are shown in Fig. 4. Owing to the uncertainty in the exact position of the laser spot with respect to the edge of the wire, the absolute values of the field strength in Fig. 4 may be between 30% lower and 75% higher, corresponding to an amplitude transmission coefficient of 20–50%.

## Discussion

We performed particle tracking simulations (General Particle Tracer^42^) to calculate the expected duration of the electron bunches at the moment they enter the tip of the wire. These simulations predict an RMS bunch length of 2.7 ps for bunches of 160 pC in the present setup. This is in reasonable agreement with the observed RMS pulse duration of the SPP of 2–3 ps. With Equation (1), we can now calculate the expected maximum electric field of the SPP. Taking an equivalent ellipsoidal electron bunch with the same RMS bunch length, this gives a peak electric field of 1.7 MV m^−1^. The measured field strength on the surface of the wire, corrected for the presence of the ZnTe crystal is 0.4 MV m^−1^ (Fig. 3b), that is, approximately a factor 4 lower than predicted for the experimental conditions used here. A possible explanation may be found if we consider the fact that the range of 3 MeV electrons in aluminium is 6 mm. The electrons therefore penetrate only the initial part of the conical tip and a significant part of the bunch is scattered into large angles, that is, sideways out of the wire. These electrons do not contribute to the CTR pulse. Furthermore, electrons that are absorbed by the wire may eject secondary electrons, which partly cancel the transition radiation field of the absorbed electrons. Nevertheless, the measured field strength is the highest reported to date measured at the surface of a metal wire. Comparison of these results to other techniques that generate SPPs directly on a wire is difficult, as the field strength is often reported in arbitrary units. In fact, we could not find any article reporting the electric field strength of SPPs on a metal wire surface.

By propagating around a 90° bend with 33 mm radius of curvature, the FWHM SPP pulse length increases from 6–10 ps to approximately 16 ps, while the maximum SPP electric field strength is reduced to 20–50% of its original value. On the basis of the measurements reported by Astley *et al*.^43^, we expected an amplitude transmission of around 15% for the bend in our setup. Our results are therefore in approximate agreement with ref. 43. The difference in path length in the *xz* plane between the inner and outer wire surface (see Fig. 2) along the 90° bend is 2.4 mm. This corresponds to 8 ps time lag of waves travelling along the outside of the bend with respect to the inside of the bend, in agreement with the observed dispersion of the SPP.

These first experiments demonstrate that intense SPPs can be excited by grazing incidence CTR, generated by ultrashort relativistic electron bunches at the conical tip of a bare metal wire. The strong fields allow quantitative analysis and comparison to the theoretical predictions made in ref. 34. The SPPs were shown to propagate around a relatively tight bend which demonstrates the potential to use these waves for THz plasmonics and the possibility to develop endoscopic THz systems. Using state-of-the-art table-top-sized accelerators^35^, extremely intense THz pulses can be created by concentrating the full THz pulse on a small tip. A particularly exciting prospect is to use the nanocoulomb, sub-100-fs, GeV electron bunches that currently drive X-FELs^25^ to excite wire-based THz SPPs. Extrapolating our measured field value using the scaling of Equation (1), such bunches should generate 0.4 GV m^−1^ THz SPPs on a millimetre-diameter wire. By tapering this wire into a sharp point^12^,^13^,^14^,^15^,^16^,^17^,^18^,^19^,^20^, these THz pulses could be delivered in a micron-sized spot, resulting in unprecedented THz field strengths of 100 GV m^−1^ and beyond.

## Methods

### SPP formation by transition radiation

By firing electron bunches onto the tip of a tapered metal wire, the radially polarized field of the electron bunches is transferred to the wire and continues to propagate along the wire as an SPP. The physical mechanism behind this process can be elucidated using the field line model for transition radiation as described by Purcell^36^. Consider an electron travelling with velocity *v* in the *z* direction, reaching the tip of a metal wire with small cone angle at time *t*=0. This situation is sketched in Fig. 1. For *t*<0, the field is that of an undisturbed charge travelling in vacuum. The thin metal wire has negligible influence because the field of the electron at relativistic speeds is confined to a thin disk perpendicular to the direction of motion. For *t*>0 when the electron is inside the metal wire, the electric fields are screened for an observer outside the metal. However, the screening is not instantaneous; instead it occurs on a sphere travelling outwards with the speed of light. Outside the sphere, indicated as region II in Fig. 1, the electric field is still that of the undisturbed charge in motion. Within the sphere, indicated as region I in Fig. 1, there is no electric field. However, the electric field lines in region II cannot abruptly end at the sphere; instead they follow the surface of the sphere ending on the metal wire where they induce a surface charge. This combination of electric fields and co-propagating surface charge is an SPP. As the electric field of an electron is very well matched to the cylindrically symmetric mode of a wire, it will excite a strong SPP. In case of an electron bunch rather than a single electron, the shell of field lines has a thickness *vτ*, where *τ* is the time it takes the bunch to pass the metal surface. We can easily calculate the electric field on the metal wire at the location of the expanding shell using Gauss's law on the closed surface indicated by the red dashed line in the figure. Because this Gauss volume does not enclose any charge, the net flux through the surface is zero. Therefore, the flux contained in the expanding sphere equals the flux crossing the Gauss volume near the surface of the wire, both being equal to *q/ɛ*_0_, with *q* the bunch charge. On the surface of the wire, this flux is spread over an annulus with radius *R* and width *cτ*. This yields the field strength given by Equation (1).

### Experimental setup

The experimental setup contains a 1.5 cell, 3 GHz radio frequency accelerator operating at a repetition frequency of 3 Hz. A Ti:Sapphire laser system produces pulses of 35 μJ at 267 nm wavelength (generated by frequency tripling of the Ti:Sapphire fundamental wavelength at 800 nm) with a duration of approximately 120 fs and a (RMS) radius of 1.5 mm. These pulses hit the copper cathode of the accelerator and generate bunches with a charge of 160 pC. The electrons are accelerated to 3.1 MeV and focussed by a quadrupole triplet onto the tip of the aluminium wire. The laser spot on the cathode is slightly elliptical, with an RMS radius of 0.1 mm in the horizontal direction and 0.15 mm in the vertical direction. At the position of the wire tip, the electron bunch is nearly circular with an RMS radius of 0.2 mm. The RMS-bunch duration at the tip of the wire, calculated by particle tracking simulations (General Particle Tracer^42^) is 2.7 ps. The wire has a radius of 0.75 mm and a conical tip with a cone angle 2*δ*=8°. The distance between the cathode surface and the tip of the wire is 1.5 m. Synchronization between the accelerator and the Ti:Sapphire laser system is better than 100 fs, using an AccTec phase-locked loop synchronizer^37^. A small part of the 800 nm laser energy (∼1 μJ) is split off before the third harmonic generation to be used as probe pulses for the electro-optic detection of the THz electric field strength *E*_*y*_ in the ZnTe crystals. These pulses pass through a linear polarizer and a *λ*/4 waveplate to make them circularly polarized. The [001] crystallographic axis of the ZnTe <110> crystal is aligned with the (horizontal) *x* axis of the setup, perpendicular to the wire. In this configuration, the electro-optic phase shift, 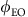, between the horizontal and vertical polarization directions is given by 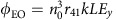, with *E*_*y*_ the electric field strength in the *y* direction, *n*_0_ the refractive index of ZnTe at 800 nm, *r*_41_ the electro-optic coefficient of ZnTe involved in the Pockels effect, *k* the wavenumber at 800 nm and *L* the thickness of the crystal. A Wollaston prism is used to separate the horizontal and vertical components of the polarization of laser pulses. The first ZnTe crystal (the one positioned before the bent section (2) of the wire, see Fig. 2) is imaged through the Wollaston prism onto two charge-coupled device cameras. The pulses passing through the second ZnTe crystal (after the bend (2) in the wire) are focussed onto two photodiodes, after passing through the prism. In both cases, the relative difference of the intensity of the signal on the two detectors is proportional to the electric field in the *y* direction: 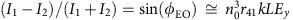, for 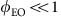, with *I*_1,2_ the intensity on each of the detectors. The thickness of both crystals is *L=*0.5 mm. For the calculation of the absolute value of the electric field strength *E*_*y*_, we have used *n*_0_=2.85 and *r*_41_=4.3 pm V^−1^ (ref. 38).

### Data availability

The data that support the findings of this study are available from the corresponding author upon request.

## Additional information

**How to cite this article:** op ‘t Root, W. P. E. M. *et al*. Single-cycle surface plasmon polaritons on a bare metal wire excited by relativistic electrons. *Nat. Commun.* **7,** 13769 doi: 10.1038/ncomms13769 (2016).

**Publisher's note:** Springer Nature remains neutral with regard to jurisdictional claims in published maps and institutional affiliations.

## Supplementary Material

Supplementary Movie 1 The movie is an animation of the experiment (with voice-over) which illustrates the method and gives a feeling for the actual dimensions of the setup.
